# The role of maternal sensitivity, infant temperament, and emotional context in the development of emotion regulation

**DOI:** 10.1038/s41598-025-01714-8

**Published:** 2025-05-19

**Authors:** Laura Bozicevic, Leonardo De Pascalis, Peter Cooper, Lynne Murray

**Affiliations:** 1 Department of Primary Care & Mental Health, University of Liverpool, Liverpool, UK; 2 School of Psychology and Clinical Language Sciences, University of Reading, Reading, UK; 3 Department of Psychology, University of Liverpool, Liverpool, UK; 4 Department of Psychology, University of Bologna, Bologna, Italy

**Keywords:** Child emotion regulation, Maternal sensitivity, Temperament, Emotional context, Observational study, Longitudinal design, Human behaviour, Emotion

## Abstract

Child emotion regulation (ER) is a multifaced system influenced by extrinsic (parenting), intrinsic (temperament) and contextual factors. Even though establishing how these factors work together is important for understanding ER developmental processes, exploration of them together has been rare, particularly in early infancy. Using a longitudinal and observational design including anger- and fear-inducing tasks, we assessed maternal sensitivity at 2–3 months (n. 144 observations) and ER at 9 months (i.e., intensity of distress, self-soothing, distraction, communicative behaviours; n. 130 observations), as well as mother-reported infant temperament. Results showed that emotional context influenced maternal sensitivity (higher in frustrating compared to novel contexts) and ER strategies (e.g., communicative behaviours were used more often when facing frustration than novelty). The effect of emotional context on ER strategies was mediated by maternal sensitivity (e.g., during frustration, higher sensitivity increased the odds of self-soothing and communicative behaviours) and moderated by temperament: greater maternal sensitivity in the context of frustration increased self-soothing in highly negative reactive children, and communicative behaviours in low reactive children. Results are discussed within ER and differential susceptibility theories to better understand ER development in early infancy and help inform effective support programmes for parents and children aimed at the prevention of emotional difficulties later in childhood.

## Introduction

### Maternal sensitivity in the context of child emotion regulation development

Sensitive caregiving—the ability to recognize infant cues and to respond promptly and appropriately to them^1^—has long been identified as a key component of parenting that promotes adaptive child development across domains of functioning, including emotion regulation^2^. Recent studies suggest that the expression of sensitivity and its impact on the child may vary according to the emotional context in which it occurs^3^. Thus, sensitivity occurring in non-distress eliciting contexts (e.g. play), and sensitivity occurring in emotionally arousing contexts (e.g. situations eliciting fear or anger) have been described as two distinctive dimensions that serve different functions in child development^4^: sensitive responses to infant non-distress cues (e.g. smiles and vocalizations occurring during, for example, play) are more strongly associated with child cognitive outcomes, whereas sensitive responses to infant distress (e.g. crying) has been found to be a unique predictor of child socio-emotional development, including emotion regulation^5,6^. However, the maternal ability to sensitively respond might itself be influenced by emotional context^7–9^: behaving sensitively in emotionally arousing situations may be particularly difficult for mothers who are vulnerable by virtue of socio-economic adversity^3,7,10^, or because of clinical conditions such as anxiety, where fear-provoking situations might be particularly challenging^11^.

To date, little research has examined the influence of emotional context on maternal sensitivity. Indeed, much of the available work has compared overall challenging vs. non-challenging situations^12^, and has neglected investigation of the effect of different emotionally-eliciting situations (e.g. frustrating and novel contexts) on parenting and later child outcomes (see for exceptions^7,11,13^). Examining the impact that specific emotional contexts have on maternal sensitivity may prove of clinical importance: difficulties shown by mothers in responding to infant’s frustration or wariness cues might translate in children’s inability to regulate specific negative emotions, such as anger and fear, later in infancy placing them at increased risk of developing externalizing and internalizing problems.

### Development of emotion regulation

The ability to modulate, inhibit, and enhance emotional experiences and expressions^14^, termed ‘emotion regulation’ (ER), is critical to the development of optimal socio-emotional, behavioural, and academic outcomes^15,16^. Children develop the foundations of their ER skills during their first year of life. In the first 3 months, infants utilize rudimentary and automatic physiological mechanisms to regulate increasingly strong emotions, and they are heavily dependent upon external sources for regulation (especially their caregivers)^17^. However, from around 6 months, they become progressively capable of intentional behaviour and of employing a range of regulatory behaviours^17^. At around 9 months there is a fundamental shift as children start using newly acquired cognitive skills that, working in concert with their developing motor skills, enable them to actively and purposefully use external resources to help manage their emotions (e.g., caregivers, objects in the environment)^16,17^. Indeed, at this age children are increasingly aware of their physical and social environment and more cognitively able to evaluate what is novel and discrepant with expectations (i.e., potentially fearful or frustrating situations), and potential sources of external support^18^.

At around nine months, when facing an emotionally challenging situation, well-regulated children are able to employ behavioural strategies which down-regulate negative affect. These include, but are not limited to, self-soothing, distraction, and making communicative bids for adult help or for their attention^19^. Although these strategies are all considered to fall within a general repertoire of regulated behaviours, previous literature suggests that particular stress-inducing contexts (such as frustration-anger, or fear-eliciting) call for *specific* strategies in order to most effectively reduce any associated distress^20–23^. For instance, in *frustrating* situations—such as when play with a toy is prevented or when social interaction is interrupted—well-regulated 9–10 month-old children are likely to use distraction (e.g., by attending to or playing with something else) and attention or communicative bids toward their parent (e.g., orienting gaze, smiling, gesturing, vocalizing)^19,22,24^. By contrast, when facing potentially fear-eliciting novel situations, well-regulated children typically look away from the source of fear, or physically step away from the situation^21,25^. Finally, self-comforting behaviours seem to be an effective strategy used by well-regulated infants in both frustrating and novel contexts^20,22^.

Although the balance of evidence suggests that children already use specific behavioural strategies in different emotionally challenging contexts during the first year of life, to date few studies have tested, in the same sample, whether the emotional context affects early child ER (reactivity and strategies) ^20,21,26^. The risk of drawing conclusions from separate studies is that what might seem to be the effect of emotional context on children’s regulatory strategies is instead the effect of individual differences between infants included in the different samples, or of variations in methodologies. This ambiguity is important to unravel because elucidation of the way in which different emotional regulation strategies are used in infancy in relation to different stress-inducing situations could provide the foundation for understanding emotion regulation in later development. This may prove of clinical importance, as the inability to regulate negative emotions, such as anger and fear, during the first year of life places children at increased risk of externalizing and internalizing problems in later infancy^15^; and it also increases the risk of developing a number of disorders later in development, such as anxiety, depression, attention deficit-hyperactivity disorder, substance use, and eating problems^16,27^.

### Factors implicated in the development of emotion regulation

The development of emotion regulation is influenced by intrinsic factors, such as temperament, and shaped by the environment, including caregivers. Temperament is defined as individual differences in emotional, motor, and attentional reactivity to external stimulation^28^. It is relatively stable over time (more so from the second, than in the first year), even though it can be susceptible to change^29^. Temperament can be assessed using standardized questionnaires completed by caregivers who indicate the level of child emotional reactivity to everyday situations (e.g., being confined in a car seat, going to sleep, being consoled, having a bath, meeting new people). Research has demonstrated that 3–10 month-old infants with a highly reactive temperament or temperamental negativity (i.e., typically showing immediate, strong, and negative responses to environmental stimulation) have poorer regulation skills compared to those who are less reactive^30,31^: they use fewer or ineffective strategies to regulate their emotions^32,33^, and are at greater risk of developing difficulties in emotion regulation later in childhood^23,34^. Indeed, frequent negative reactions to the environment shown by high emotionally reactive children might hinder their exploration and their employment of a wider range of ER strategies. In the case of fearful children, they might miss out on opportunities for new experiences such as acquiring new information, learning new skills, or meeting new people, thereby putting them at higher risk of developing anxiety^35^. Similarly, anger-prone children might miss opportunities for learning because they may attempt to avoid what they perceive as restrictions posed by the environment, take longer to reorganize their behaviours, and have a shorter attention span^33^, placing them at a greater risk of developing externalizing problems^15,16^.

Despite the findings supporting the association between temperament and emotion regulation, recent research posits that the effect of temperament on child development is influenced by factors such as the caregiving environment^29^. Thus, as well as biological mechanisms, ER development is influenced by social partners, particularly in the first 2–3 months of life when infants rely heavily on external sources for the regulation of their emotions^17^. Indeed, sensitive parenting, in particular maternal sensitivity in the context of potential stress, as highlighted above, is likely to calm the infant’s physiological state^36^ and influence physiological regulation^37^, thereby facilitating the development of future ER skills^38,39^. Although both temperament and parenting can exert direct effects on child ER, accumulating evidence suggests an interplay between these factors in influencing child development, although the precise relationship between them remains to be determined. A recent line of research, following Belsky’s differential susceptibility theory, supports the idea that temperament moderates the association between maternal sensitivity and child development^40^. According to this theory, compared to temperamentally low reactive children, children who are highly reactive are particularly susceptible to variations in the environment, and their development suffers in the context of insensitive caregiving; by contrast, their outcomes are particularly favourable when the caregiving they receive is of high quality. On this view, the influence of maternal sensitivity on child ER development is moderated by the child’s temperament. However, most of the studies following Belsky’s differential susceptibility theory have looked at the interaction between temperament and maternal sensitivity in the prediction of attachment security and behavioural problems, with little focus on ER outcomes (for exceptions see^30,31,34,41^). To our knowledge, the few studies which looked at the interaction between maternal sensitivity and infant temperament in predicting ER found that high maternal sensitivity in the context of better temperament (e.g. low/medium surgency or low negative reactivity) fosters adaptive ER (e.g., more frequent use of attentional strategies, fewer dysregulatory behaviours)^30,31,34^. However, in these studies maternal sensitivity was assessed either as average of behaviours observed in different tasks^31^ or in not emotionally challenging tasks (e.g. play)^30,34^, neglecting to explore the effect that the emotional context (i.e., novel, frustrating) might have on both the maternal ability to respond sensitively to the child and the child’s capacity to regulate their own emotions (in terms of reactivity and ER strategies).

**Fig. 1:**
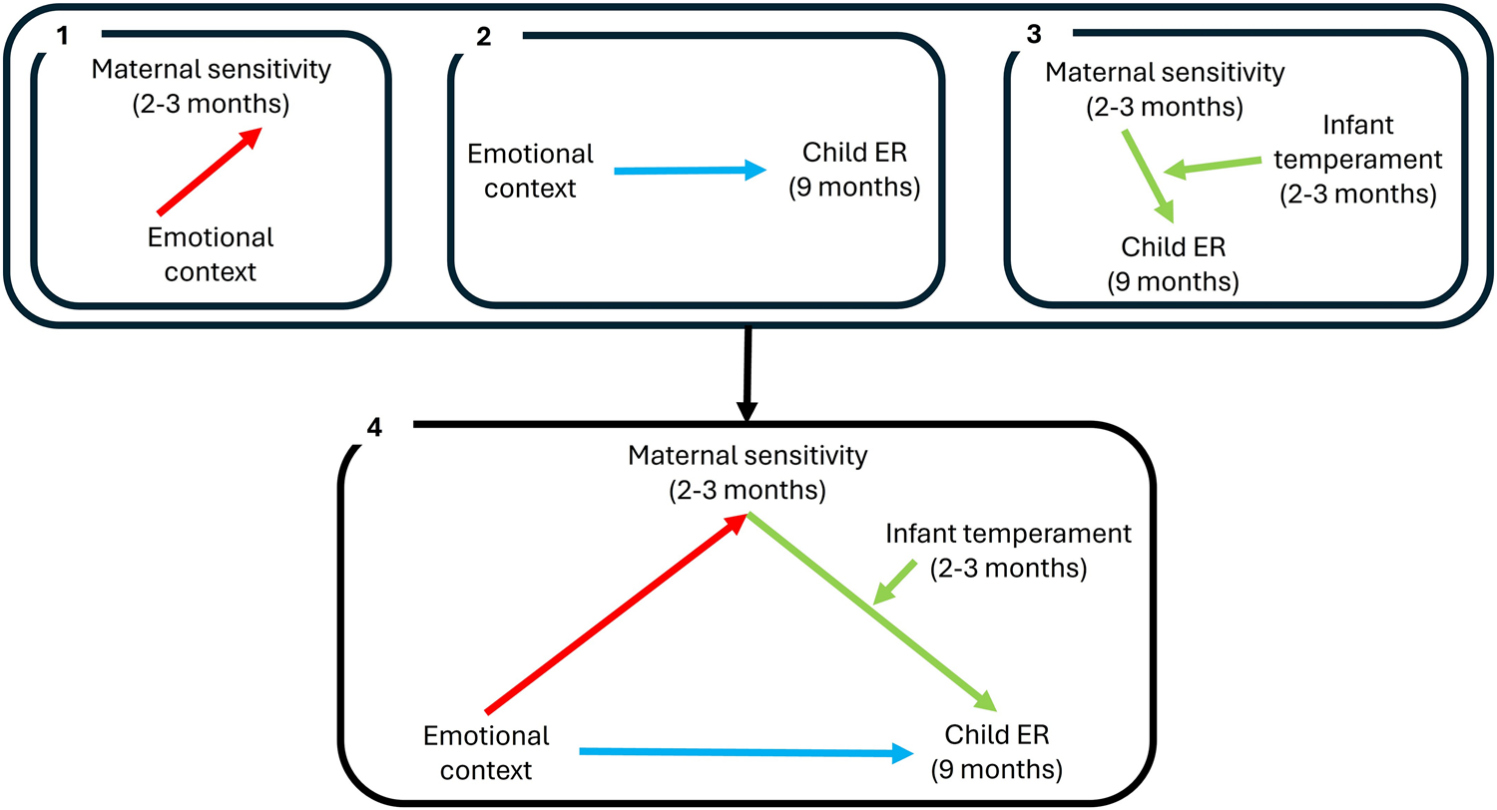
Predicted relationships.

In summary, we propose a model whereby emotional context, infant temperament and parenting operate together to affect the development of child emotion regulation, as set out in Fig. 1. We tested this model in a longitudinal study with infants aged 3–9 months and their caregivers, and examined the following specific predictions: (1) that differences in maternal sensitivity to infants at 2–3 months (time 1) would be found between emotional contexts (frustrating vs. novel). No specific direction for these differences was hypothesised because of limited and mixed literature available; (2) that differences in observed child ER (i.e., intensity of distress, self-soothing, distraction and communicative behaviours) at 9 months (time 2) would be found between emotional contexts. In accordance with previous research, we predicted more frequent infant use of distraction and communicative strategies in frustration contexts compared to novelty contexts, while no differences were expected in the intensity of distress and use of self-soothing strategies^10,22,42^; (3) that maternal sensitivity at 2–3 months would be associated with child ER at 9 months, and further that this relationship would be moderated by infant temperament. Specifically, and line with the differential susceptibility theory, we predicted that only in children with high negative reactivity, would there be a positive association between maternal sensitivity and child emotion regulation (i.e., lower intensity of distress and more frequent use of regulated strategies); (4) following on from the three previous predictions, we hypothesised that maternal sensitivity at 2–3 months would mediate the association between emotional context and child emotion regulation, taking the moderating role of infant temperament into account. That is, variation in child emotion regulation between emotional contexts at 9 months would be explained by earlier context-dependent variations in maternal sensitivity, accounting for the above-mentioned moderation of infant temperament.

## Results

### Data analyses

Given the repeated measurements (frustration measured in two tasks vs. novelty measured in two tasks), a generalized mixed model framework was adopted, varying the underlying distribution assumptions, based on the specific outcome variable.

Differences between emotional contexts were investigated for maternal sensitivity, using a gaussian distribution with an identity link, and for child ER strategies, assuming a Poisson distribution, with log link, and task time as offset. A similar Poisson model was used when testing whether infant temperament moderated the relationship between maternal sensitivity and child ER strategies, controlling for the effect of frustration vs. novelty. Because the same effects were multiply tested on different outcome variables in the models described above (all performed in R^43^), the *p*-values reported below, related to child ER, were corrected using the Bonferroni method. For all reported Generalised Linear Mixed Models effects, marginal R^2^ values are reported, as calculated using the R “partR2” package. Power analysis was based around the most complex model tested (with emotional context, and the main and interactive effects of maternal sensitivity and child temperament predicting child ER behaviour rate); a simulation approach, using the R “simr” package^44^ was adopted (the syntax is included in the Supplementary Materials), assuming a medium effect size in the relationship between maternal sensitivity and emotional context and child behaviour, and a small to medium effect size of 0.2 change in the rate of child ER behaviours per unit change in the predictors. A power of 0.80 was estimated to be achievable with a sample size of 34, given the specific design tested in the current study (each participant tested 4 times, for an estimated total of 136 observations). A power plot—Figure S1—is included in the Supplementary Materials, showing change in power at different simulated sample sizes. Mediation and moderated mediation models were conducted using the R “mediation” package^45^ and bias-corrected and accelerated (BCa) bootstrapping (with 5000 resamplings) to estimate confidence intervals of indirect effects^46^. While, in the proposed mediation model, mediator and outcome are assessed at two different time points, predictor and mediator are measured concurrently, which may appear to create uncertainty around direction of effects. Here, however, the predictor variable is a tested condition (i.e., frustration vs. novelty), unamenable to the effect of maternal sensitivity, rendering the effect of emotional context on maternal sensitivity the only reasonably investigable one. A *p*-value < 0.05 was considered significant.

### Descriptives

The thirty-eight participants were generally at low socio-economic risk, had full-term pregnancies, with infant birthweight > 2.5 kg. Mothers were British (all but three were of white ethnicity, two were mixed-Black and one mixed-Asian) and had completed at least high school education. The great majority were in a stable relationship (all but two participants were living with their husband or partner). None of the socio-demographic characteristics was significantly correlated with child ER, and so were not controlled for in the analyses. Depression and anxiety scores measured at 2–3 months assessment (time 1) as potential covariates were, on average, low (Depression: 4.43 (*SD* 3.221); Anxiety: 3.30 (*SD* 2.518)). Since depression and anxiety were potentially of direct relevance to maternal sensitivity and infant ER, we examined their association, but, in this low-risk sample, results were not significant.

Table 1 shows mean scores and standard deviations of maternal sensitivity scores at time 1 (n = 144 observations), and infant intensity of distress and emotion regulation strategies (i.e., self-soothing, distraction and communicative behaviours) at time 2 (n = 130 observations). Tables showing these same variables, but in the four different emotional contexts (specifying number of participants and score ranges in each), are included in the Supplementary Materials (Tables S1 and S2). The overall mean negative emotionality score was 3.55 (*SD* 0.704), ranging from 2.32 to 5.31; the mean scores for children with low emotional reactivity and high emotional reactivity were 2.94 (*SD* 0.317) and 4.15 (*SD* 0.391), respectively.

**Table 1:** Descriptives—maternal sensitivity at 2–3 months, and child emotion regulation at 9 months.

	Mother (2–3 months)		Child (9 months)		
	Maternal sensitivity (scale 0–5)	Intensity of distress (scale 0–5)	Soothing (%)	Distraction (%)	Communicative behaviours (%)
	M (SD)	M (SD)	M (SD)	M (SD)	M (SD)
Frustration	3.63 (1.01)	1.52 (1.37)	3.87 (7.39)	17.81 (13.72)	3.69 (3.97)
Novelty	3.32 (0.83)	1.69 (2.12)	3.93 (7.52)	4.10 (5.65)	2.58 (3.82)

### The effect of emotional context on maternal sensitivity

A significant difference in maternal sensitivity was found between emotional contexts (frustration vs. novelty) (*F*(1.000, 106.624) = 6.341, *p* = 0.013, *R*^2^ = 0.027), with higher sensitivity shown by mothers in contexts of frustration compared to novelty.

### The effect of emotional context on child emotion regulation

No significant difference between emotional contexts was found in the intensity of child distress (*F*(1, 95.792) = 0.272, corrected *p* > 0.999, *R*^2^ = 0.002), or in the rate per minute of child self-soothing behaviours (${X}^{2}$(1) = 3.687, corrected *p* = 0.219, *R*^2^ = 0.012). A significant difference was found in the rate per minute of child distraction behaviours (${X}^{2}$(1) = 1018.480, corrected *p* < 0.001, *R*^2^ = 0.226), and of child communicative behaviours (${X}^{2}$(1) = 10.810, corrected *p* = 0.004, *R*^2^ = 0.042), which were both more frequent in the context of frustration, than novelty.

### The effect of maternal sensitivity on child emotion regulation, and the moderating role of child temperament

The association between maternal sensitivity and emotion regulation was tested, controlling for emotional context (frustration vs. novelty). Maternal sensitivity was not associated with the intensity of child distress (*F*(1, 121) = 0.075, corrected *p* > 0.999, *R*^2^ < 0.001), but was instead significantly associated with the rate of child ER strategy use: the more sensitive the mother, the more frequent the use of child self-soothing behaviours (${X}^{2}$(1) = 31.366, corrected *p* < 0.001, *R*^2^ = 0.009) and communicative behaviours (${X}^{2}$(1) = 27.654, corrected *p* < 0.001, *R*^2^ = 0.029), and the less frequent the use of distraction in children (${X}^{2}$(1) = 10.253, corrected *p* = 0.005, *R*^2^ = 0.001).

Early child negative reactivity was not found to moderate the effect of maternal sensitivity on the rate per minute of distraction behaviours (${X}^{2}$(1) = 3.385, corrected *p* = 0.197, *R*^2^ = 0.009), but it did moderate its effect on child self-soothing behaviours (${X}^{2}$(1) = 61.205, corrected *p* < 0.001, *R*^2^ = 0.008) and communicative behaviours (${X}^{2}$(1) = 15.013, corrected *p* < 0.001, *R*^2^ = 0.039).

While maternal sensitivity was found to be negatively associated with the rate of self-soothing behaviours in children with low negative reactivity (*b* = − 0.195, 95% BCa CI = − 0.379 to − 0.015, *p* = 0.033), a positive relation emerged in the group of highly reactive children (*b* = 0.789, 95% BCa CI = 0.615–0.971, *p* < 0.01). Maternal sensitivity was also found to be positively associated with the rate of communicative behaviours in children with low negative reactivity (*b* = 0.763, 95% BCa CI = 0.514–1.030, *p* < 0.01), while no significant association was found in the high negative reactive group (*b* = 0.149, 95% BCa CI = − 0.046 to 0.346, *p* = 0.131).

### Maternal sensitivity as a mediator of the effects of emotional context on child emotion regulation, and the moderating role of child temperament

Having found an association between sensitivity and ER strategies, mediation analyses were conducted to test whether the variation in child emotion regulation between emotional contexts (e.g., more frequent use of self-soothing and communicative behaviours in response to frustration compared to novelty) was explained by the fact that mothers were more sensitive in the context of frustration compared to novelty.

#### Child self-soothing behaviours

The lack of difference in the use of self-soothing behaviours between frustration and novelty contexts previously described appeared to be the result of two competing and contrasting effects: on the one hand, frustrating contexts presented lower rates of these behaviours, compared to novelty contexts (*b* = − 0.012, 95% BCa CI = − 0.026 to − 0.002, *p* = 0.024), as shown by the significant direct effect of emotional context on child self-soothing behaviours when accounting for sensitivity; on the other hand, and apparently cancelling out this direct effect in a case of inconsistent mediation, frustration was also associated with an increase in sensitivity, in turn associated with an increase in the rate of self-soothing behaviours (*b* = 0.007, 95% BCa CI = 0.001–0.016).

The indirect effect of emotional context on child self-soothing via maternal sensitivity was found to change according to the level of early child emotional reactivity: for all children, frustration contexts presented higher maternal sensitivity than in novelty contexts and this increase was associated with more frequent child self-soothing in highly emotionally reactive children (*b* = 0.018, 95% BCa CI = 0.002–0.044), and with fewer of such behaviours in children with low emotional reactivity (*b* = − 0.006, 95% BCa CI = − 0.019 to 0.00003).

#### Child distraction

In another case of inconsistent mediation, the indirect effect of emotional context on the rate per minute of child distraction behaviours, via maternal sensitivity (*b* = − 0.008, 95% BCa CI = − 0.019 to − 0.001), acted as suppressor of the direct effect of emotional context on these child behaviours: while frustrating contexts presented more use of distraction in children, they were also associated with higher maternal sensitivity, in turn associated with lower frequency of child distraction behaviours. Unsurprisingly, when taking this role of sensitivity into account, the direct effect of frustration on distraction remained significant (*b* = 0.275, 95% BCa CI = 0.217–0.342, *p* < 0.001).

#### Child communicative behaviours

An indirect effect of emotional context on child communicative behaviours through maternal sensitivity was found (*b* = 0.007, 95% BCa CI = 0.001–0.015): contexts of frustration presented higher maternal sensitivity, which was in turn associated with increased child communicative behaviour rates. The direct effect of emotional context on child communicative behaviours nonetheless remained significant (*b* = 0.015, 95% BCa CI = 0.005–0.027, *p* = 0.003). The indirect effect of emotional context on child communicative behaviours via maternal sensitivity was found to change according to the level of early child emotional reactivity, with the indirect pathway only found to be significant in children with low early negative reactivity (*b* = 0.012, 95% BCa CI = 0.002–0.029).

## Discussion

The present longitudinal observational study aimed to explore how differences in emotional context, maternal sensitivity and infant temperament assessed at 2–3 months contribute to child emotion regulation assessed at 9 months. Following recent studies suggesting that maternal sensitivity occurring when children are in emotionally arousing contexts (e.g., eliciting fear or anger) might be a salient predictor of child socio-emotional development^5,6,13^, we designed developmentally appropriate tasks which varied according to their emotion eliciting properties. This design allowed us to gather novel information on how variation in emotional context relate to both maternal sensitivity to infant signals in the first 2–3 months and ER at 9 months, and how maternal sensitivity mediates the association between emotional context and ER. In addition, we assessed the moderating role of child temperament, as an intrinsic factor influencing emotional development, in the association between maternal sensitivity and child ER, in line with Belsky’s differential susceptibility theory^39,40^

Findings from the current study support the hypothesis that parenting and child ER behaviours are susceptible to contextual characteristics, and that mothers and children adjust to the demands of different situations^9,23,27,47^. On one hand, levels of sensitivity were higher in the context of infant frustration than when infants were confronted by novelty, despite infant distress being comparable in the two conditions. On the other hand, when facing frustration children more frequently used distraction and communicative behaviours compared to in novel contexts, while no differences emerged in the use of self-soothing, in line with our hypotheses and previous studies^20,22,24^.

Research on the role of maternal sensitivity, assessed in the first year in the context of child distress, suggests that high sensitivity is associated with better child ER, both concurrently and in later development^5,6^. However, empirical data supporting this association are scarce, especially in relation to early infancy and comparing different emotion-eliciting contexts. Present findings supported the hypothesis that variation in child emotion regulation between emotional contexts is explained by context-dependent variations in maternal sensitivity: greater maternal sensitivity in the context of frustration increased the rate of self-soothing and communicative behaviours at 9 months when facing frustration. Both these strategies are adaptive and successfully employed by infants to regulate distress, with communication being considered more mature, and being more frequently used later in development ^17,48^. However, sensitivity did not mediate the association between emotional context and intensity of distress, possibly because this reactive component of ER is similar to a temperamental trait, not as susceptible to external influences as the regulative component (i.e., ER strategies).

When looking at the association between maternal sensitivity, and self-soothing and communicative strategies, infant temperament assessed at 2–3 months appeared to play an important moderating role. In line with Belsky’s differential susceptibility theory (2005)^40^, we hypothesized that only in the group of highly negative reactive children there would be a positive association between maternal sensitivity and child emotion regulation. Our results were only partially consistent with such theory: maternal sensitivity observed during frustration tasks was indeed associated with better ER skills in the highly negative reactive group of children, expressed through the higher use of self-soothing. However, this association was found to be significant, but negative for the low reactive children who also benefitted from high maternal sensitivity, showing greater use of communicative behaviours as a strategy in the face of frustration (an association that was not present in highly reactive children). This latter finding, not aligned with Belsky’s theory, might be related to our examination of specific strategies to regulate emotions, rather than more global measures of ER, with this specificity enabling the detection of pathways between parenting and ER, according to infant temperament, that are not typically explored within this theoretical framework. Moreover, it should be noted that Belsky’s differential susceptibility theory has been tested mainly in relation to attachment^49^, and internalizing and externalizing problems^50,51^. A limited number of previous studies have focused on emotional regulation, reaching differing conclusions. Frick and colleagues’ (2018) found that high levels of early maternal sensitivity at 10 months promoted ER development at 18 months only in the group of children with low-to-medium levels of ‘surgency’ (i.e., better regulated)^31^, consistent with our findings that sensitive mothers fostered the use of communicative strategies in the low-reactive group of children. The study conducted by Bolten and colleagues (2013) found that 6 month-old infants with high neonatal reactivity were likely to show good emotion regulation capacities (i.e., higher number of self-quieting behaviours employed during the Still-Face paradigm) when maternal cortisol reactivity to a stressor were low (i.e., considered as index of optimal parenting environment), in line with our result on sensitive mothers promoting self-soothing behaviours in the negatively reactive children group^41^. By contrast, Gunning and colleagues (2013) found that behavioural dysregulation assessed in the recovery period of the Still-face was lower for neonatally irritable infants of highly sensitive mothers (compared to non-irritable infants of either sensitive or insensitive mothers), whereas they did not find evidence to suggest that irritable neonates of highly sensitive mothers were better regulated than their non-irritable peers (as one should expect according to differential susceptibility theory)^30^. Our findings seem to indicate that sensitive mothers of highly reactive children recognise that an important goal for their children to be able to regulate frustration is to lower their physiological activation and their levels of arousal, with self-soothing being the ideal strategy to achieve this^17^. This hypothesis is in line with research indicating that infant negative emotionality (NE) has different effects on maternal sensitivity based on the mothers’ context^52^. Specifically, low-SES mothers tend to show lower sensitivity in the context of high infant NE, while high-SES mothers show higher sensitivity to high NE infants^52^. The theory is that mothers who are more well-resourced may be able to recognize that their infants need more from them, and they work with the infant’s temperament rather than against it. As this was a sample of highly educated, well-resourced women, it is possible that mothers of highly reactive children may recognize that their children need more help accomplishing the goal of learning self-soothing behaviours. Our findings, together with the ones from the literature described, indicate the need for further studies on the relationship between sensitivity, temperament, and specific aspects of child ER.

Although our study sample was a low-risk group, it is worth considering possible clinical implication of our findings. The fact that, even in this low-risk sample, early maternal sensitivity and emotional context were relevant to subsequent child ER, highlights the potential importance of intervening early in supporting mothers and infants, taking into consideration both infant temperamental characteristics and specific aspects of the emotional context that might hinder the maternal ability to foster infants’ optimal socio-emotional development. Indeed, some clinical interventions with families have used a domain-based theory, where interactions between individuals are seen as being organized according to different domains^8^. When parents can be helped to support their children’s emotion regulation according to the relevant domain (e.g., attachment domain vs. exploration domain), the child can develop appropriate behaviour and expression of emotions, and the resources needed to deal with challenges, thereby mitigating the risk of developing emotional problems^8^. Therefore, it might be worth assessing parenting in different contexts other than play, as this might elucidate difficulties not otherwise revealed, as suggested by both this domains-based theory, and work examining domain-specific variations in the effect of maternal depression on child outcomes^53^.

Strengths of this study are the longitudinal and observational design, including assessments of maternal sensitivity and infant temperament, and of child ER in two different conditions. This design enabled the exploration of the effects of variations in these external and internal factors on ER. Moreover, as most studies on ER have been conducted during toddlerhood, the early exploration of emotional context, sensitivity, and temperament allowed for a contribution to the understanding of the early origins of ER development. Despite these strengths, some limitations should be considered. The observations of child ER strategies were brief due to the nature of the tasks and the age of the children. Another limitation was the small sample size. While a sample size adequate for power requirements was met for several outcomes, this number was only approximated, albeit very closely, for some measures due to some missing observations. If future studies were able to replicate the design used in our research with a larger sample, this would allow an analysis of different temperamental aspects (e.g., distress to limitation, fearfulness) in relation to regulatory behaviours shown in the different emotion-eliciting contexts. Further, in addition to assessing global aspects of parenting such as sensitivity, future studies could examine specific maternal behaviours (e.g., maternal distracting, soothing, talking) which could directly influence later regulatory strategies employed by children. Moreover, the potential role of parent individual differences on their behaviour in each context could not be tested in the present study because there was not enough variability in depression and anxiety scores or in educational background; in addition, other parenting-related variables were not assessed, such as personality traits or socio-economic status. Future studies should include the assessment of individual differences in caregivers, not only related to their mental health (e.g. depression, anxiety, personality), but also related to their ethnicity and socio-economic conditions. Finally, in addition to assessing ER competences, later socio-emotional outcomes (e.g., compliance, behavioural inhibition, externalizing problems), which have been demonstrated to be associated with infants’ ability to regulate emotions, could also be assessed to better understand pathways of development and individuate areas of risk and intervention.

To conclude, the results of this research highlight that it is essential to take several factors into account in order to understand infant emotion regulation and its development. Notably, while maternal behaviour and infant temperament have long been considered in the study of emotional development, the role of the emotional context in which the emotions are regulated has been a neglected topic. Equally importantly, above and beyond considering each of these factors as independent influences, our findings highlight that their interplay is crucial: the kind of contextual challenge shapes the mother’s responsive offer, which ultimately affects how the infant will regulate their emotions in that situation, based on their temperament. Therefore, it emerges that the emotional context influences the shape of the social exchange, which influences the developmental outcome, which is moderated by individual’s characteristics and resources.

## Methods

### Participants

Thirty-eight UK mother-infant dyads participated in the present study.

Mothers were recruited from the postnatal ward of the Royal Berkshire Hospital (Reading, UK), a general community maternity hospital, and all provided informed consent. The socio-demographic characteristics of the sample are presented in Table 2. The study was conducted according to the British Psychological Society’s Code of Human Research Ethics and approved by the Ethics Committee of the University of Reading (n. 14/35).

**Table 2:** Demographic information.

	N	Mean (SD)	Range
Maternal age	38	32.74 (4.963)	19.00–42.00
Gestational age (weeks)	38	40.11 (1.178)	37.00–42.00
Birth weight (kg)	38	3.61 (0.415)	2.81–4.93
Child age—time 1	38	2.93 (0.165)	2.50–3.30
Child age—time 2	34	9.43 (0.300)	9.05–10.20

	N	%	
Child sex (male)	22	57.9	
Parity (primiparous)	24	63.2	
Ethnicity (white)	35	92.1	
Education (degree or higher)*	26	68.4	
Marital status (married/cohabiting)	36	94.7	

*In the UK having a degree means obtaining either a General Certificate of Secondary Education (GCSEs)/Ordinary Level (O-levels) or an Advanced Level (A-level) or a National Vocational Qualification (NVQ), or a Higher National Diploma (HND) or equivalents qualifications.

### Procedure

Mothers were approached on the postnatal ward and asked whether they would be interested in taking part in developmental research being conducted at the University of Reading. Mothers who agreed to take part were assessed at multiple timepoints. Data reported in the current paper include assessments conducted at 2–3 months (time 1) and 9 months (time 2). During each visit, mothers were asked to fill in questionnaires on their mood and on child temperament. Filming of maternal and child behaviour during five tasks (described in detail below) was conducted in two different potentially emotionally challenging contexts, frustration and novelty (see Table 3). Maternal sensitivity was assessed at time 1 during the reunion phase of the Still-face procedure and the Arm restraint task (frustrating context), and during the Stranger approach and the New toy tasks (novel context); data on child emotion regulation was assessed at time 2 during the still-face phase of the Still-face procedure and the Toy removal task (frustrating context), and during the Stranger approach and the New toy tasks (novel context) (see Table 3). Tasks were interrupted if the infant became highly distressed for more than 10 s, in which case mothers were free to soothe their children.

**Table 3:** Assessment contexts for ratings of maternal sensitivity and child ER in frustration versus novelty contexts.

	Frustration		Novelty	
Maternal sensitivity (time 1)	Still face	Arm restraint	Stranger approach	New toy
Child ER (time 2)	Still face	Toy removal	Stranger approach	New toy

### Frustration

#### Still face

The Still-Face Procedure (SFP) is a standard laboratory procedure used for the evaluation of infant emotion regulatory strategies and interactive characteristics of the mother-infant dyad, by assessing the infant’s response to violations of expected social responsiveness (i.e., caregiver stopping their interaction with the child)^54^. Even though the SFP was originally theorised within the field of early social interactions to study infants’ communicative abilities, in the past 10–20 years the task has been used as a context of frustration to investigate children’s physiological and behavioural reaction and management of negative emotions caused by the interruption of the communication by a parent. This type of frustration is similar to the one that children may experience on a daily basis (e.g., when parents are unable to respond despite infant’s expectation of and desire for a response)^47,55–57^.

The SFP, used at time 1 and 2, comprised three episodes: a face-to-face play episode when the caregiver and child were free to interact; a still-face episode, during which the caregiver did not respond to the infant while holding a neutral expression; and a reunion episode, when the caregiver resumed interacting with her infant and manages any distress emerged in the previous phase. Lengths for each episode vary across studies^58^; in the present research, the free interaction episode lasted 3 min, the still-face 1.5 min, and the reunion 2 min.

#### Arm restraint

The Arm Restraint procedure^59^ is a task resembling moments of ordinary physical restraint caused, for instance, by the use of seatbelts or by being confined in a crib and was used at time 1 as the frustrating task. The infants were seated in a semi-reclined position, with the researcher seated in front of them (with their head bowed so as to minimize interaction with the infants), gently holding the infants’ forearms down to their sides for 1.5 minutes^20,59^. Mothers were seated in view of the infants and free to interact whilst the researcher was holding the infants’ forearms.

#### Toy removal

As the arm restraint task is not developmentally appropriate to elicit frustration at 9 months, a Toy Removal task was used instead as frustrating context at time 2^59^. Children were left to play with a standard set of toys provided by the researcher on a mat on the floor, while their mother filled in questionnaires nearby; after 3 min of play, the researcher removed the toys placing them in view of the children, but out of reach for 1 min, after which the toys were given back to the child to play with for a further 2 min. The 1-min episode of Toy Removal was used to assess child’s ER.

### Novelty

#### Stranger approach

The Stranger Approach is a task designed to assess children’s emotional reaction to a socially novel situation. The procedure, used in the present study at time 1 and 2, was adapted from Goldsmith (1999) and Murray and colleagues (2007)^11,59^, and included two episodes. In the first episode (2 min), the children were seated in an infant seat, with their mothers sitting next to them and instructed to refrain from interacting, and a researcher unknown to the mother and the child gradually approached the child following a scripted procedure (calling child’s name and saying a few words, coming closer and talking to the child, touching the child’s feet and cheeks, and finally picking up the child). This episode was used to assess child ER when children were 9 months (time 2). In the second episode (1 min) the researcher, after sitting the child back in their seat, started talking to the mother (similarly following a script to ensure consistency across mothers); during this episode, the mother was free to interact with the infant as she wished, while talking to the stranger. This episode of the task was used to code maternal sensitivity when infants were 2–3 months (time 1), in line with previous literature which assessed maternal behaviour during the Stranger Approach task at 10 weeks of infant age^11^.

#### New toy

The New Toy task was used as a non-social novel situation during time 1 and 2. At first, children were left to explore a novel toy by themselves for 3 min, and then mothers were encouraged to play with them using the toy provided for 1.5 min. Toys used during time 1 and 2 were age appropriate and differed between the two visits, but they had in common properties such as making noises and moving parts, which made them unpredictable, and acoustically and visually stimulating: when infants were 2–3 months (visit 1), we used a little piano (suspended over infants head while they were reclined on their back) which was playing music, flashing lights and moving randomly with jerky movements; when children were 9 months (visit 2), we used a “roll & chase bumble bee ball” which was rolling unpredictably and randomly making noises and playing music while flashing lights. The first episode (children playing by themselves) was used to assess child ER at 9 months, while the second (mothers encouraging infants to interact with the toy when 2–3 months)) was used to assess maternal sensitivity.

### Measures

#### Socio-demographic questionnaire.

Demographic information was collected during the first visit, including maternal age, parity, ethnicity, education, and marital status, and infant sex, gestational age, and weight at birth. Age of the child was collected at each visit (Table 2).

#### Maternal depression and anxiety symptomatology

Symptoms of maternal depression and anxiety were assessed at time 1 (2–3 months) using the Hospital Anxiety and Depression Scale^60^, a 4-point scale questionnaire including fourteen items and extensively used in non-psychiatric hospital clinics and research. The HADS has an Anxiety subscale (HADS-A) and a Depression subscale (HADS-D), both including seven items. The higher are the aggregated scores in the two scales, the higher is the level of mood disturbance. It is suggested that a score < 7 indicates no depression or anxiety, while a score ≥ 8 indicates possible depression/anxiety^60^. The scale has acceptable internal consistency (for the anxiety items the correlations range from 0.76 to 0.41; for the depression items the correlations range from 0.60 to 0.30) and good convergent validity (correlations between interview ratings and patient ratings are 0.54 for the anxiety scale and 0.79 for the depressive symptoms scale).

#### Maternal sensitivity

Maternal sensitivity was assessed at time 1 (2–3 months) during each of the four emotionally eliciting tasks described in the previous section using the Sensitivity scale from the Global Rating Scales of mother-infant interaction (GRS^61^). Mothers were rated according to their ability to respond promptly and appropriately to infant cues and emotional states, for instance commenting on infants’ interest, following their gaze, empathizing with them, and soothing them when distressed, imitating or responding to their vocalizations in a positive way. Maternal sensitivity was rated by a trained coder on a 5-point scale (higher scores indicating higher sensitivity).

#### Child temperament

Child temperament was assessed at time 1 (2–3 months) using the Infant Behaviour Questionnaire—Revised Short Form (IBQ-R Short Form^62^), a 91-item questionnaire which has been found to be reliable in assessing temperament characteristics throughout infancy^63^. Mothers were asked to indicate, on a 7-point scale, how frequently during the previous week their infant responded to specific events (e.g., when confined in a car seat or in a crib, or when exposed to a loud noise or a new person) by fussing, crying, or with no negative reaction. The Negative Emotionality scale was selected for the present study and includes a sum of four subscales: Distress to Limitations (distress to frustrating events), Fear (distress to novelty), Sadness (general low mood), and Falling Reactivity (reversed scored recovery from peak distress, excitement, or general arousal). The higher are the scores obtained on this scale, the higher is the temperamental negativity. Infants were divided into low and high emotionally reactive groups using a median split, in line with the method used by De Rosnay and colleagues^64^. The IBQ-R Short Form has shown good internal consistency (Cronbach’s alpha for the Negative Emotionality factor equalling 0.91).

#### Child emotion regulation

Child Emotion Regulation was assessed at time 2 (9 months) during the four emotion-eliciting tasks described using a coding scheme developed by Bozicevic and colleagues^15,38^. ER was assessed on two distinct dimensions which reflect child reactivity and regulatory behaviours:

(a) *Intensity of distress:* distress shown by the children through negative facial expressions and negative vocalizations (including crying) were rated using an overall score for each of the four tasks; scores ranged from 0 (no distress shown for the whole duration of task) to 5 (escalated crying at any point during the task or prolonged distress).

(b) *ER strategies:* behaviours displayed by children during each task were rated second-by-second as mutually exclusive categories and are reported in the analyses as percentage of task duration (i.e., for each task, percentage of task time spent using each behaviour); ER strategies included are:

- Self-soothing (e.g., thumb-sucking, self-hug, hair-curling, hands clasping, eye-rubbing, and proximity-seeking);
- Distraction (e.g., attending to or manipulating an object, other than the stimulus, for example playing with or exploring their own clothes, their shoes, part of the seat such as the seatbelt, or the mat if seated on the floor);
- Communicative behaviours (e.g., trying to make social contact for example pointing, vocalizing and smiling toward the mother or the researcher).

**Reliability.** Twenty percent of the videos were coded, independently, by two trained English-speaking researchers. The first author acted as gold standard for the coding used and trained two other researchers (blind to the purpose of the study and to the relevant variables assessed – i.e., socio demographic characteristics, maternal depression and anxiety symptomatology, child temperament); after reaching reliability, one researcher coded maternal sensitivity, while the other coded child emotion regulation of all the remaining videos. Interclass correlations was 0.85 for maternal sensitivity and Cohen’s Kappa ranged between 0.71 and 0.95 for child emotion regulation codes (i.e., intensity of distress, self-soothing, distraction, and communicative behaviours).

## Supplementary Information

Supplementary Information.

## Data Availability

The research data is archived in the University of Liverpool repository (Data Catalogue DOI: 10.17638/datacat.liverpool.ac.uk/2750).

## References

[1] Ainsworth, M. D. S., Bell, S. M. & Stayton, D. J. Infant–mother attachment and social development. In *The Introduction of the Child into a Social World* (ed. Richards, M. P.) 99–135 (Cambridge University Press, 1974).

[2] Rocha, N. A. C. F. et al. Impact of mother–infant interaction on development during the first year of life: A systematic review. *J. Child Health Care* **24**, 365–385 (2020). 31337225 10.1177/1367493519864742

[3] Taraban, L., Shaw, D. S., Morris, P. A. & Mendelsohn, A. L. An exploration of the domain specificity of maternal sensitivity among a diverse sample in the infancy period: Unique paths to child outcomes. *Child Dev.* **95**, e60–e73 (2024). 37612891 10.1111/cdev.14000 PMC11549955

[4] Leerkes, E. M., Weaver, J. M. & O’Brien, M. Differentiating maternal sensitivity to infant distress and non-distress. *Parenting* **12**, 175–184 (2012). 22798728 10.1080/15295192.2012.683353 PMC3393126

[5] Leerkes, E. M., Blankson, A. N. & O’Brien, M. Differential effects of maternal sensitivity to infant distress and nondistress on social-emotional functioning. *Child Dev.* **80**, 762–775 (2009). 19489902 10.1111/j.1467-8624.2009.01296.x PMC2854550

[6] Conradt, E. & Ablow, J. Infant physiological response to the still-face paradigm: contributions of maternal sensitivity and infants’ early regulatory behavior. *Infant Behav. Dev* **33**, 251–265 (2010). 20207419 10.1016/j.infbeh.2010.01.001

[7] Mills-Koonce, W. R. et al. Infant and parent factors associated with early maternal sensitivity: A caregiver-attachment systems approach. *Infant Behav. Dev* **30**, 114–126 (2007). 17292784 10.1016/j.infbeh.2006.11.010

[8] Hill, J. et al. The application of a domains-based analysis to family processes: Implications for assessment and therapy. *J. Fam. Ther.* **36**, 62–80 (2014).

[9] Miller, A. L., McDonough, S. C., Rosenblum, K. L. & Sameroff, A. J. Emotion regulation in context: Situational effects on infant and caregiver behavior. *Infancy* **3**, 403–433 (2002).

[10] Leerkes, E. M. et al. Antecedents of maternal sensitivity during distressing tasks: Integrating attachment, social information processing, and psychobiological perspectives. *Child Dev.* **86**, 94–111 (2015). 25209221 10.1111/cdev.12288 PMC5242093

[11] Murray, L., Cooper, P., Creswell, C., Schofield, E. & Sack, C. The effects of maternal social phobia on mother–infant interactions and infant social responsiveness. *J. Child Psychol. Psychiatry* **48**, 45–52 (2007). 17244269 10.1111/j.1469-7610.2006.01657.x

[12] Dittrich, K. et al. Observational context of mother-child interaction: Impact of a stress context on emotional availability. *J Child Fam Stud* **26**, 1583–1591 (2017).

[13] Leerkes, E. M. Maternal sensitivity during distressing tasks: A unique predictor of attachment security. *Infant Behav. Dev* **34**, 443–446 (2011). 21616538 10.1016/j.infbeh.2011.04.006 PMC3134119

[14] Calkins, S. D. & Hill, A. Caregiver influences on emerging emotion regulation: biological and environmental transactions in early development. In *Handbook of emotion regulation* (ed. Gross, J. J.) 229–248 (The Guilford Press, 2007).

[15] Bozicevic, L. et al. Longitudinal association between child emotion regulation and aggression, and the role of parenting: A comparison of three cultures. *Psychopathology* **49**, 228–235 (2016). 27486811 10.1159/000447747 PMC5659189

[16] Stifter, C. A. & Augustine, M. Emotion regulation. In *Handbook of Emotional Development* (eds LoBue, V. et al.) 405–430 (Springer, 2019).

[17] Kopp, C. B. Antecedents of self-regulation: A developmental perspective. *Dev. Psychol.* **18**, 199–214 (1982).

[18] Scherer, K. R., Zentner, M. R. & Stern, D. Beyond surprise: The puzzle of infants’ expressive reactions to expectancy violation. *Emotion* **4**, 389–402 (2004). 15571437 10.1037/1528-3542.4.4.389

[19] Stifter, C. A., Spinrad, T. L. & Braungart-Rieker, J. Toward a developmental model of child compliance: the role of emotion regulation in infancy. *Child Dev.* **70**, 21–32 (1999). 10191513 10.1111/1467-8624.00003

[20] Buss, K. & Goldsmith, H. H. Fear and anger regulation in infancy: Effects on the temporal dynamics of affective expression. *Child Dev.* **69**, 359–374 (1998). 9586212

[21] Diener, M. L. & Mangelsdorf, S. C. Behavioral strategies for emotion regulation in toddlers: Associations with maternal involvement and emotional expressions. *Infant Behav. Dev* **22**, 569–583 (1999).

[22] Stifter, C. A. & Braungart, J. M. The regulation of negative reactivity in infancy: Function and development. *Dev. Psychol.* **31**, 448–455 (1995).

[23] Roque, L., Veríssimo, M., Fernandes, M. & Rebelo, A. Emotion regulation and attachment: Relationships with children’s secure base, during different situational and social contexts in naturalistic settings. *Infant Behav. Dev* **36**, 298–306 (2013). 23542812 10.1016/j.infbeh.2013.03.003

[24] Braungart-Rieker, J. M. & Stifter, C. A. Infants’ responses to frustrating situations: Continuity and change in reactivity and regulation. *Child Dev.* **67**, 1767–1779 (1996). 8890506

[25] Crockenberg, S. C. & Leerkes, E. M. Infant and maternal behavior moderate reactivity to novelty to predict anxious behavior at 2.5 years. *Dev. Psychopathol.* **18**, 17 (2006). 16478550 10.1017/S0954579406060020

[26] Leerkes, E. M. & Wong, M. S. Infant distress and regulatory behaviors vary as a function of attachment security regardless of emotion context and maternal involvement. *Infancy* **17**, 455–478 (2012). 22919285 10.1111/j.1532-7078.2011.00099.x PMC3422878

[27] Cole, P. M., Michel, M. K. & Teti, L. O. D. The development of emotion regulation and dysregulation: A clinical perspective. *Monogr. Soc. Res. Child Dev.* **59**, 73–102 (1994). 7984169

[28] Rothbart, M. K. & Derryberry, D. Development of individual differences in temperament. In *Advances in Developmental Psychology* (eds Lamb, M. E. & Brown, A. L.) 37–86 (Erlbaum, 1981).

[29] Shiner, R. L. et al. What is temperament now? Assessing progress in temperament research on the twenty-fifth anniversary of Goldsmith et al. *Child Dev. Perspect.* **6**, 436–444 (2012).

[30] Gunning, M., Halligan, S. L. & Murray, L. Contributions of maternal and infant factors to infant responding to the Still Face paradigm: a longitudinal study. *Infant Behav. Dev* **36**, 319–328 (2013). 23548574 10.1016/j.infbeh.2013.02.003

[31] Frick, M. A. et al. The role of sustained attention, maternal sensitivity, and infant temperament in the development of early self-regulation. *Br. J. Psychol.* **109**, 277–298 (2018). 28895129 10.1111/bjop.12266

[32] Stifter, C. A. & Spinrad, T. L. The effect of excessive crying on the development of emotion regulation. *Infancy* **3**, 133–152 (2002). 33451205 10.1207/S15327078IN0302_2

[33] Calkins, S. D., Dedmon, S. E., Gill, K. L., Lomax, L. E. & Johnson, L. M. Frustration in infancy: Implications for emotion regulation, physiological processes, and temperament. *Infancy* **3**, 175–197 (2002). 33451201 10.1207/S15327078IN0302_4

[34] Thomas, J. C., Letourneau, N., Campbell, T. S., Tomfohr-Madsen, L. & Giesbrecht, G. F. Developmental origins of infant emotion regulation: Mediation by temperamental negativity and moderation by maternal sensitivity. *Dev. Psychol.* **53**, 611 (2017). 28333524 10.1037/dev0000279

[35] Buss, K. A. Which fearful toddlers should we worry about? Context, fear regulation, and anxiety risk. *Dev. Psychol.* **47**, 804 (2011). 21463035 10.1037/a0023227 PMC3086967

[36] Porges, S. W. & Furman, S. A. The early development of the autonomic nervous system provides a neural platform for social behaviour: A polyvagal perspective. *Infant Child Dev.* **20**, 106–118 (2011). 21516219 10.1002/icd.688 PMC3079208

[37] Perry, N. B., Calkins, S. D. & Bell, M. A. Indirect effects of maternal sensitivity on infant emotion regulation behaviors: The role of vagal withdrawal. *Infancy* **21**, 28–153 (2016). 10.1111/infa.12101 PMC4806398 27019648

[38] Bozicevic, L. et al. Sculpting culture: Early maternal responsiveness and child emotion regulation—A UK-Italy comparison. *J. Cross-Cult. Psychol.* **52**, 22–42 (2021).

[39] Sroufe, L. A. *Emotional Development: The Organization of Emotional Life in the Early Years* (Cambridge University Press, 1996).

[40] Belsky, J. Differential susceptibility to rearing influence. In *Origins of the Social Mind: Evolutionary Psychology and Child Development* (eds Ellis, B. & Bjorklund, D.) 139–163 (Guilford Press, 2005).

[41] Bolten, M. et al. Prenatal programming of emotion regulation: Neonatal reactivity as a differential susceptibility factor moderating the outcome of prenatal cortisol levels. *J. Psychosom. Res.* **75**(4), 351–357. 10.1016/j.jpsychores.2013.04.014 (2013). 10.1016/j.jpsychores.2013.04.014 24119942

[42] Braungart-Rieker, J. M., Hill-Soderlund, A. L. & Karrass, J. Fear and anger reactivity trajectories from 4 to 16 months: The roles of temperament, regulation, and maternal sensitivity. *Dev. Psychol.* **46**, 791 (2010). 20604602 10.1037/a0019673

[43] R Core Team. *R: A Language and Environment for Statistical Computing* (R Foundation for Statistical Computing, 2024).

[44] Green, P. & MacLeod, C. J. SIMR: An R package for power analysis of generalized linear mixed models by simulation. *Methods Ecol. Evol.* **7**, 493–498 (2016).

[45] Tingley, D., Yamamoto, T., Hirose, K., Keele, L. & Imai, K. Mediation: R package for causal mediation analysis. *J. Stat. Softw.*10.18637/jss.v059.i05 (2014).

[46] Hayes, A. F. Beyond Baron and Kenny: Statistical mediation analysis in the new millennium. *Commun. Monogr.* **76**, 408–420 (2009).

[47] Blacher, J., Baker, B. L. & Kaladjian, A. Syndrome specificity and mother–child interactions: Examining positive and negative parenting across contexts and time. *J. Autism Dev. Disord.* **43**, 761–774 (2013). 22829243 10.1007/s10803-012-1605-x PMC3548024

[48] Ekas, N. V., Lickenbrock, D. M. & Braungart-Rieker, J. M. Developmental trajectories of emotion regulation across infancy: Do age and the social partner influence temporal patterns. *Infancy* **18**, 729–754 (2013). 10.1111/infa.12003 PMC3828040 24244107

[49] Cassidy, J., Woodhouse, S. S., Sherman, L. J., Stupica, B. & Lejuez, C. Enhancing infant attachment security: An examination of treatment efficacy and differential susceptibility. *Dev. Psychopathol.* **23**, 131–148 (2011). 21262044 10.1017/S0954579410000696

[50] Pluess, M. & Belsky, J. Differential susceptibility to rearing experience: The case of childcare. *J. Child Psychol. Psychiatry* **50**, 396–404 (2009). 19175816 10.1111/j.1469-7610.2008.01992.x

[51] Bradley, R. H. & Corwyn, R. F. Infant temperament, parenting, and externalizing behavior in first grade: A test of the differential susceptibility hypothesis. *J. Child Psychol. Psychiatry* **49**, 124–131 (2008). 18211274 10.1111/j.1469-7610.2007.01829.x

[52] Taraban, L. & Shaw, D. S. Parenting in context: Revisiting Belsky’s classic process of parenting model in early childhood. *Dev. Rev.* **48**, 55–81 (2018).

[53] Murray, L., Cooper, P. & Fearon, P. Parenting difficulties and postnatal depression: Implications for primary healthcare assessment and intervention. *Community Pract.* **87**, 34–38 (2014). 25612413

[54] Tronick, E., Als, H., Adamson, L., Wise, S. & Brazelton, T. B. The infant’s response to entrapment between contradictory messages in face-to-face interaction. *J. Am. Acad. Child Adolesc. Psychiatry* **17**, 1–13 (1978). 10.1016/s0002-7138(09)62273-1 632477

[55] Gago-Galvagno, L. G. et al. The still-face paradigm in Latin American mother–child dyads at 2 and 3 years: Effects of socioeconomic status and temperament. *J. Exp. Child Psychol.* **217**, 105357 (2022). 35066419 10.1016/j.jecp.2021.105357

[56] Hill, A. L. & Braungart-Rieker, J. M. Four-month attentional regulation and its prediction of three-year compliance. *Infancy* **3**, 261–273 (2002). 33451200 10.1207/S15327078IN0302_9

[57] Qu, J. & Leerkes, E. M. Patterns of RSA and observed distress during the still-face paradigm predict later attachment, compliance and behavior problems: A person-centered approach. *Dev. Psychobiol.* **60**, 707–721 (2018). 29797313 10.1002/dev.21739

[58] Mesman, J. et al. The many faces of the Still-Face Paradigm: A review and meta-analysis. *Dev. Rev.* **29**, 120–162 (2009).

[59] Goldsmith, H. & Rothbart, M. K. *The Laboratory Temperament Assessment Battery (Lab-TAB): Pre-locomotor Version 3.1* (Department of Psychology, University of Oregon, 1999).

[60] Zigmond, A. S. & Snaith, R. P. The hospital anxiety and depression scale. *Acta Psychiatr. Scand.* **67**, 361–370 (1983). 6880820 10.1111/j.1600-0447.1983.tb09716.x

[61] Murray, L., Fiori-Cowley, A., Hooper, R. & Cooper, P. The impact of postnatal depression and associated adversity on early mother-infant interactions and later infant outcome. *Child Dev.* **67**, 2512–2526 (1996). 9022253

[62] Rothbart, M. K. Measurement of temperament in infancy. *Child Dev.* **52**, 569–578 (1981).

[63] Dias, C. C., Costa, R., Pinto, T. M. & Figueiredo, B. The infant behavior questionnaire–revised: Psychometric properties at 2 weeks, 3, 6 and 12 months of life. *Early Hum. Dev.* **153**, 105290 (2021). 33316587 10.1016/j.earlhumdev.2020.105290

[64] De Rosnay, M., Cooper, P. J., Tsigaras, N. & Murray, L. Transmission of social anxiety from mother to infant: An experimental study using a social referencing paradigm. *Behav. Res. Ther.* **44**, 1165–1175 (2006). 16288978 10.1016/j.brat.2005.09.003

