# Supplementary material for: The role of maternal sensitivity, infant temperament, and emotional context in the development of emotion regulation
Source: Sci Rep. 2025 May 19;15:17271. doi: 10.1038/s41598-025-01714-8 (PMC12089384; doi:10.1038/s41598-025-01714-8)

Supplementary Materials

Tables

Table S1. Descriptives - Maternal sensitivity and infant temperament at 2-3 months

	Maternal Sensitivity			Negative Emotionality		
	N	M (sd)	Range	N	M (sd)	Range
Total	144	3.47 (0.931)	1-5	38	3.55 (0.704)	2.32-5.31
Still Face (Frustration)	38	3.55 (0.957)	1-5			
Arm Restraint (Frustration)	33	3.73 (1.069)	2-5			
Stranger Approach (Novelty)	35	3.20 (0.797)	2-5			
New Toy (Novelty)	38	3.42 (0.85)	1.5-5			

Table S2. Descriptives – Child Emotion Regulation at 9 months

	Intensity of Distress (scale 0-5)			Soothing (%)			Distraction (%)			Communicative behaviours (%)		
	N	M (sd)	Range	N	M (sd)	Range	N	M (sd)	Range	N	M (sd)	Range
Total	130	1.61 (1.789)	0-5	130	6.50 (12.379)	0.00-71.76	130	17.90 (20.687)	0.00-86.67	130	5.19 (6.528)	0.00-30.00
Still Face (Frustration)	30	1.70 (1.317)	0-4	30	4.73 (10.085)	0.00-44.12	30	40.72 (20.226)	0.00-86.67	30	6.12 (6.366)	0.00-25.56
Toy Removal (Frustration)	33	1.36 (1.410)	0-5	33	8.02 (14.002)	0.00-66.67	33	19.64 (20.591)	0.00-71.67	33	6.17 (6.941)	0.00-28.33
Stranger Approach (Novelty)	34	2.82 (2.263)	0-5	34	10.98 (16.007)	0.00-71.67	34	11.16 (10.955)	0.00-34.17	34	2.88 (5.068)	0.00-23.33
New Toy (Novelty)	33	0.52 (1.093)	0-4	33	1.97 (4.181)	0.00-15.83	33	2.37 (4.316)	0.00-19.17	33	5.75 (7.253)	0.00-30.00

Power Analysis Syntax

```
if (!require("pacman"))
  install.packages("pacman")
pacman::p_load(simr, tidyverse)

power_plot <-
tibble(
  n = numeric(),
  power = numeric(),
  lower = numeric(),
  upper = numeric()
)

for(i in c(2, 13, 24, 34, 45, 56, 67, 78, 89, 100)) {
set.seed(123456)

n <- i

simdata <-
  tibble(
    id = rep(1:n, each = 4),
    f_n = rep(c(0, 1), each=2, length.out = n * 4),
    neg = rep(c(0, 1), each = 4, length.out = n * 4),
    sens_a = (0.3 * f_n) + (0.3 * neg) + rnorm(n = n * 4, mean = 0, sd = 1))

fixef_f_n <- log(1.2)
fixef_sens_a <- log(1.2)
fixef_neg <- log(1.2)
fixef_sens_a_neg <- log(0.8)
```

```
fixef_int <- log(5)

model <-
  makeGlmer(
    y ~ f_n + sens_a + neg + sens_a:neg + (1 | id),
    family="poisson",
    fixef = c(fixef_int, fixef_f_n, fixef_sens_a, fixef_neg, fixef_sens_a_neg),
    VarCorr = 0.5,
    data = simdata)

sim <- powerSim(model, nsim = 1000, test = fixed("sens_a:neg"), seed = 123456)
power_plot <-
  power_plot |>
  bind_rows(
    c(
      n = i,
      power = (sim |> summary() |> as.data.frame() |> pull(mean)) * 100,
      lower = (sim |> summary() |> as.data.frame() |> pull(lower)) * 100,
      upper = (sim |> summary() |> as.data.frame() |> pull(upper)) * 100
    )
  )
}

p_plot <-
  power_plot %>%
  ggplot(aes(y = power / 100, x = n, ymin = lower / 100, ymax = upper / 100)) +
  geom_line(colour = 'red') +
  geom_errorbar(width = 1.5) +
  geom_point() +
```

```
  geom_hline(yintercept = 0.8, color = 'gray70', lty = 2) +
  geom_vline(xintercept = 34, color = 'blue', lty = 2) +
  scale_y_continuous(name = 'Power', limits = c(0, 1),
                     breaks = c(0, .1, .2, .3, .4, .5, .6, .7, .8, .9, 1),
                     labels = c('0%', '10%', '20%', '30%', '40%', '50%', '60%', '70%', '80%', '90%',
'100%')) +
  scale_x_continuous(name = 'Number of participants',
                     breaks = c(0, 10, 20, 30, 40, 50, 60, 70, 80, 90, 100)) +
  theme_bw() +
  theme(axis.title.x = element_text(size = 16),
        axis.title.y = element_text(size = 16),
        axis.text = element_text(size = 12))

ggsave("Power.png", p_plot, width = 12, height = 6, units = "in", dpi = 300)
```

Figure S1. Power Plot (the blue dashed line corresponds to a sample size of 34)

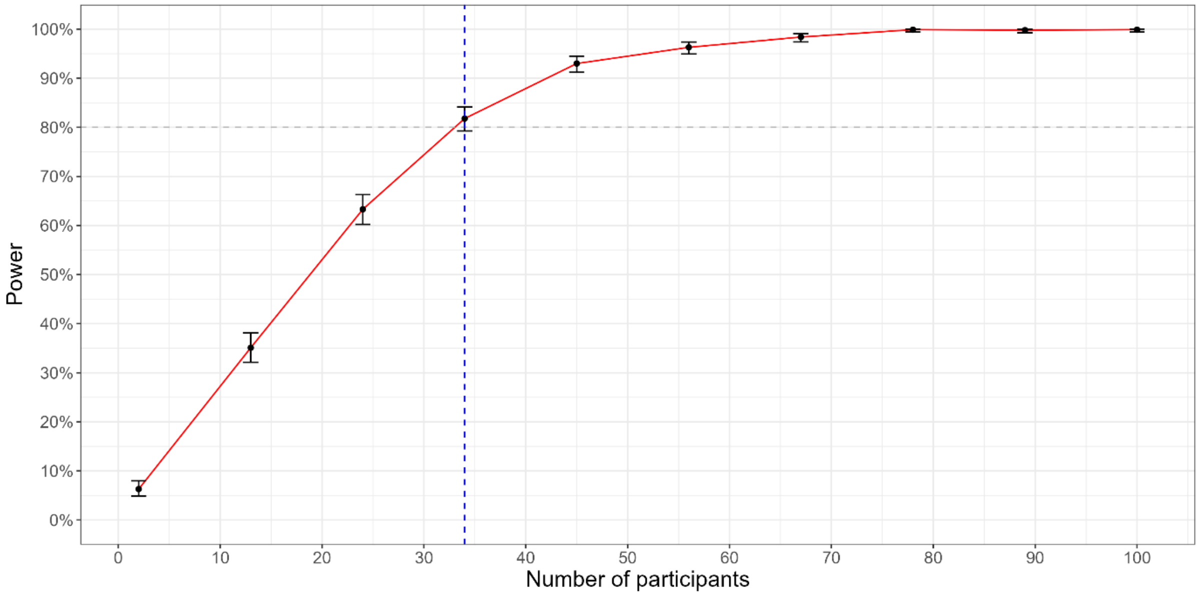

Supplement: Supplementary file 1 — Supplementary Information. [file 41598_2025_1714_MOESM1_ESM.pdf]
